# The EPICURE study: a pilot prospective cohort study of heterogeneous and massive data integration in metastatic breast cancer patients

**DOI:** 10.1186/s12885-021-08060-8

**Published:** 2021-03-31

**Authors:** Mathilde Colombié, Pascal Jézéquel, Mathieu Rubeaux, Jean-Sébastien Frenel, Frédéric Bigot, Valérie Seegers, Mario Campone

**Affiliations:** 1 Scientific and innovation department, ICO Integrated Center for Oncology, Bd J. Monod, 44805 Nantes, Saint Herblain France; 2 Nuclear medicine unit, ICO Integrated Center for Oncology, Bd J. Monod, 44805 Nantes, Saint Herblain France; 3 Bioinfomic unit, ICO Integrated Center for Oncology, Bd J. Monod, 44805 Nantes, Saint Herblain France; 4 Keosys, 13 Impasse Serge Reggiani, 44815 Saint-Herblain, France; 5 Medical oncology department, ICO Integrated Center for Oncology, Bd J. Monod, 44805 Nantes, Saint Herblain France; 6 CRCINA, INSERM, CNRS, Université d’Angers, Université de Nantes, Nantes, France; 7 Medical oncology department, ICO Integrated Center for Oncology, 15 Rue André Boquel, 49055 Angers, France; 8 Biometric unit, ICO Integrated Center for Oncology, Bd J. Monod, 44805 Nantes-, Saint Herblain, France

**Keywords:** Metastatic breast cancer, Cohort, Prediction, Collection, Heterogeneous, Multi-omics data, Integration, Quality of life, Molecular imaging, Drug resistance, Return to work

## Abstract

**Background:**

Breast cancer is the most common cancer in women and the first cancer concerning mortality. Metastatic breast cancer remains a disease with a poor prognosis and about 30% of women diagnosed with an early stage will have a secondary progression. Metastatic breast cancer is an incurable disease despite significant therapeutic advances in both supportive cares and targeted specific therapies. In the management of a metastatic patient, each clinician follows a highly complex and strictly personal decision making process. It is based on a number of objective and subjective parameters which guides therapeutic choice in the most individualized or adapted manner.

**Methods/design:**

The main objective is to integrate massive and heterogeneous data concerning the patient’s environment, personal and familial history, clinical and biological data, imaging, histological results (with multi-omics data), and microbiota analysis. These characteristics are multiple and in dynamic interaction overtime. With the help of mathematical units with biological competences and scientific collaborations, our project is to improve the comprehension of treatment response, based on health clinical and molecular heterogeneous big data investigation.

**Discussion:**

Our project is to prove feasibility of creation of a clinico-biological database prospectively by collecting epidemiological, socio-economic, clinical, biological, pathological, multi-omic data and to identify characteristics related to the overall survival status before treatment and within 15 years after treatment start from a cohort of 300 patients with a metastatic breast cancer treated in the institution.

**Trial registration:**

ClinicalTrials.gov identifier (NCT number): NCT03958136. Registration 21st of May, 2019; retrospectively registered.

## Background

### Disease background

Breast cancer is the most common cancer in women with 58,459 new cases in France in 2018. It is the first cancer concerning mortality with 12,146 deaths in 2018, but mortality rate is decreasing in France since the last 15 years. This decreasing rate is in relation with early detection, screening and adjuvant therapies [1].

Metastatic breast cancer remains a disease with a poor prognosis with a 5-year survival less than 20%, and a median-survival of 24 to 30 months after metastasis diagnosis. Each year 5 to 10% of new breast cancers are diagnosed with a metastatic staging. About 30% of women diagnosed with an early stage will have a secondary progression. Metastatic breast cancer is an incurable disease despite significant therapeutic advances in both supportive cares and targeted specific therapies (anti-HER2, anti-estrogenic) and cytotoxic molecules [2–5]. This therapeutic arsenal improves clearly quality of life of patients, and sometimes a gain in terms of overall survival.

### General management of therapies in metastatic breast cancer

In the management of a metastatic patient, each clinician builds his own decision algorithm. It is based on a number of objective and subjective parameters which allow the therapeutic decision making process to become the most individualized or adapted:

- Extrinsic objectives parameters are currently based on EBM (evidence-based-medicine): the age of the patient, the aggressiveness of the disease, previous therapies (neoadjuvant, metastatic), relapse time to initial diagnosis, hormone receptor (HR) expression, estrogen (ER +) and / or progesterone (PR +), overexpression of the oncogene HER2 (HER2 +), mutation of PIK3CA, ESR1or BCRA1/2, expression of PDL1 and previous clinical trial results (overall survival, time to progression).
- Intrinsic subjective parameters are taken into account in decision-making: parameters that are linked to the oncologist’s assumptions, such as, for example, the sensitivity to the theoretical efficacy of treatments and the definition of sensitivity. From the point of view of the patient, the choice is influenced by her more or less pregnant social life, the experience of a previous treatment, her age, her psychological state, her symptoms and the survival hoped gain.

### Current therapeutic strategies

Currently, the clinician rationalizes these therapeutic indications according to the prediction of the treatment response from the “phenotypic classification” [6–8]. This immunohistochemistry (IHC)-based classification includes three subtypes: breast cancers defined as luminal by HR positivity, HER2 + cancers and triple-negative cancers (HR and HER2 -). The targeting of oncogenic addictive pathways by anti-estrogenic therapies (SERM - Selective Estrogen Receptor Modulators, SERD - Selective Estrogen Receptor Degradation and aromatase inhibitors) or HER2 inhibitory approaches (trastuzumab, pertuzumab, TDM-1,lapatinib, neratinib) induces mitigate signals of death, survival, and cell proliferation [9–11]. However, initially, the signals of death and cellular arrest are predominant and then they reverse under therapeutic pressure. The tumor escapes by adapting to its new environment induced by the treatment.

The identification of resistance or adaptive pathways led to the development of additive strategies. This strategy with a strong EBM literature has been shown to be effective in both ER + (CDK, mTOR and PI3Kinase inhibitors) and HER2 + patients (pertuzumab, lapatinib, TDM-1, neratinib) [12]. This strategy derives directly from the first strategy, via the identification (by DNA sequencing technique) of anomalies for which there is a specific therapy [13–15]. However, the decision algorithms, described and based on a target-one treatment, are not optimal and it is now necessary to define a new therapeutic strategy based on a systemic approach for a complex disease.

### Research hypothesis

**Fig. 1:**
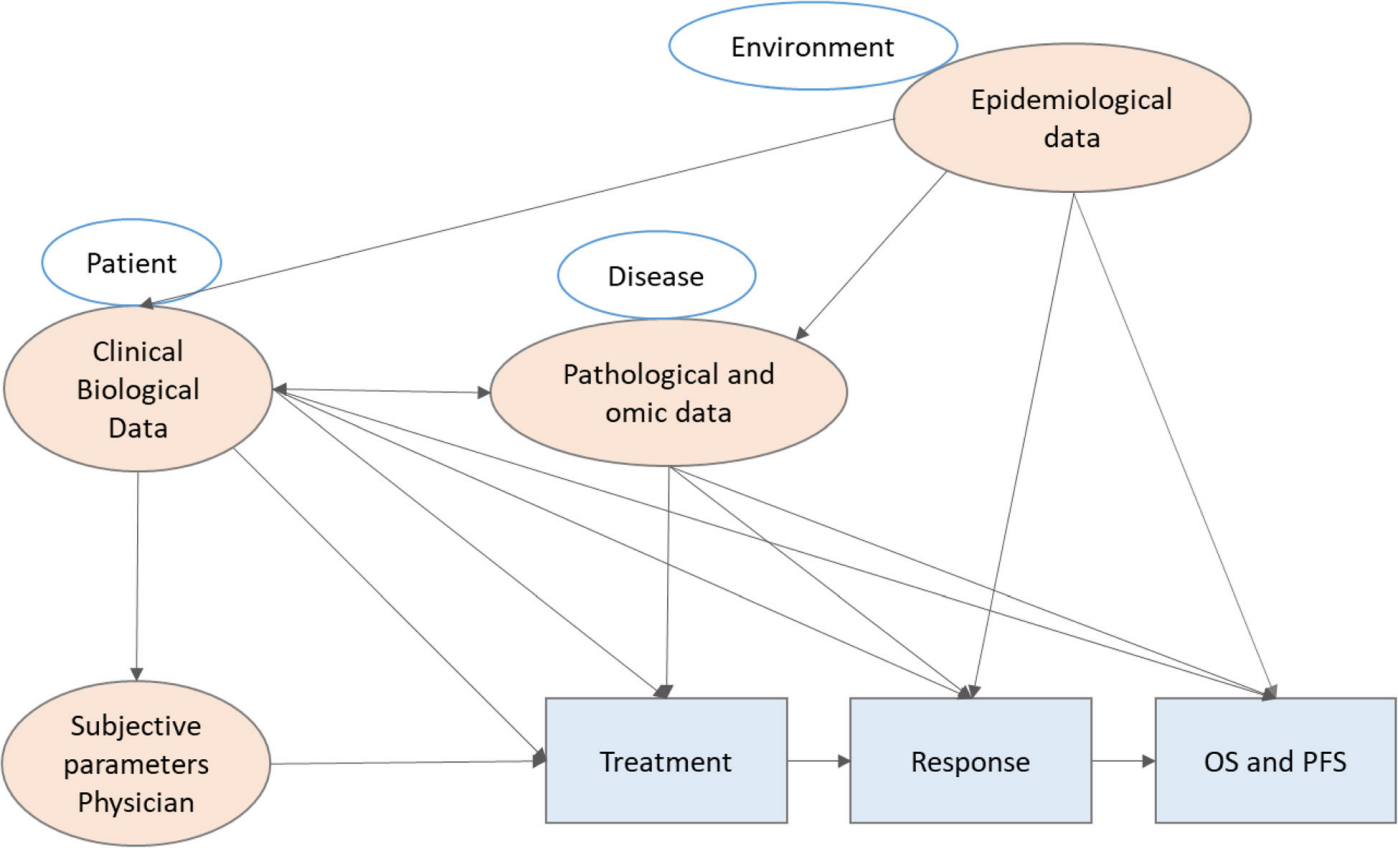
Underlying links and systemic approach

As explained above, current therapeutic strategies are based on a reductionist approach, and they do not meet the expected success. Cancer is a complex disease relying on multiple parameters in dynamic, organized and evolving interactions, and analysis of a complex system requires a systemic approach (Fig. 1).

Thus, we need to evolve from a reductionist, disjunctive, analytical view of the characterization of cell components (genes, transcripts, proteins, etc.) to a global, systemic, conjunctive and organizational vision: distinct datasets are linked and we need to unravel these underlying links.

### Massive data

In our current and modern clinical practice with new innovative and numeric tools, physicians collect massive data relative to the patient. Multi-omics approach is now described in literature [16–18].

In a global approach it seems important to collect the most exhaustive global information about the patient and not only the biological characteristics. However, these data are usually heterogeneous, quantitative versus qualitative, possibly censored or missing.

To our knowledge, little literature exists about the exploitation of such massive and heterogeneous data in metastatic breast cancer field.

We thus intend to integrate a massive and curated database with dynamic data overtime that will allow us to model the metastatic cancer during its various stages of progression, and will help us to understand it and better individualize the treatments.

Nevertheless, the heterogeneity, the censured character of the data, and above all, the very large number of variables with respect to the number of patients involve the use of statistical methods which have the ability to remain efficient despite these constraints (see the mathematical section below for details). As a consequence, it seems of first importance to associate the expertise of several teams in order to provide a satisfying method to decide which treatment process is the most adapted to each patient.

### Rationale for conducting this study

Resistance to treatment in metastatic breast cancer remain poorly understood. The hypothesis on the multifactorial mechanisms of resistance must include tumor datas, patients and environment datas and need to be prospectively studied. This hypothesis explains the building of this prospective database concerning metastatic breast cancer patients.

This database contains epidemiological, socio-economic, clinical, biological, imaging, pathological and multi-omics data in order to take into account this multifactorial hypothesis.

With this project, we want to demonstrate the ability to exploit complex data in healthcare and in particular in cancer management. We chose a specific metastatic breast cancer model with no literature available for mathematical development in this application field. By sharing dynamic expertise in massive data and mathematics with different units, we want to enhance therapeutic management in the actual metastatic breast cancer example chosen.

Justification for this study is based on the following 3 points:

- Prediction and new modelling of breast cancer outcome from complex data sources
- Creation of algorithms and expertise to use massive data in cancer management
- Interdisciplinary databases and co-working for data collection and analysis.

### Study objectives

#### Short term and main purpose

To prove feasibility of creation of a clinico-biological database prospectively by collecting epidemiological, socio-economic, clinical, biological, imaging, pathological and multi-omics data before treatment and within 15 years after treatment start.

At the end of feasibility period, this database will contain a complete view for 300 patients. Once proved the feasibility, further prospective inclusions will permit in the mid-term to identify with sufficient statistical power the independent prognostic parameters for 15 yr-overall survival among the environmental, clinical, biological, imaging, bio-pathological and omic collected characteristics of patients with metastatic breast cancer.

#### The secondary objectives are

- To describe response to treatment for each therapeutic sequence and identify new prognostic and predictive factors related to treatment response (assessed by multi-omic biological and tumor tissue analysis).
-To evaluate progression free survival (PFS) for each therapeutic sequence.
- To identify the predictive factors of resistance to treatment.
- To identify new prognostic and predictive factors related to treatment response.
- To describe socio demographic profile, quality of life, Emotional Vulnerability, physical activity practice, nutritional assessment before and after treatments.

#### Exploratory objective

We will use in silico methods to integrate together complex data (epidemiological characteristics, clinical, biological, imaging, bio-pathological, and microbiota characteristics of each patient [19]) from this cohort in order to define an algorithm of individual decision for the prediction of the treatment response with needs to develop new statistical and modeling tools.

## Design

### Study design

**Fig. 2:**
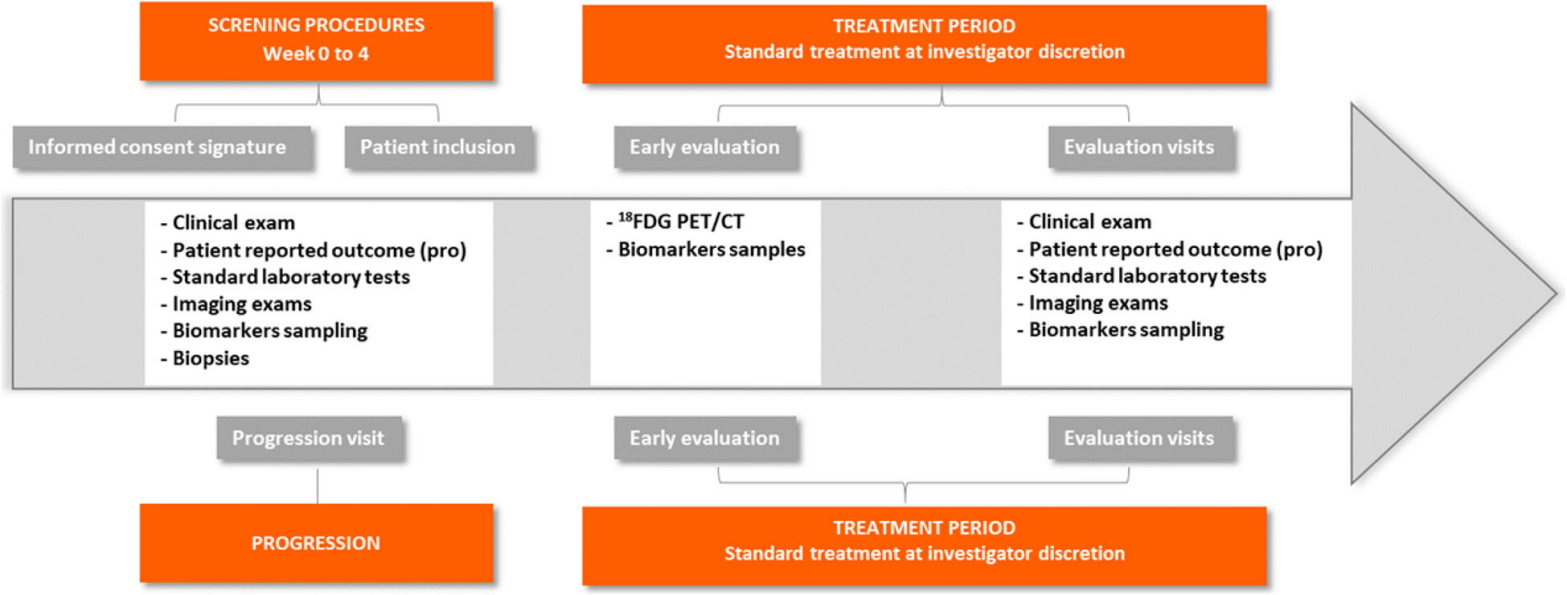
study design

This is a prospective uncontrolled cohort study of patients with metastatic breast cancer (Fig. 2).

Patients are followed in the institution (ICO cancer center, Nantes and Angers) with the usual therapeutic care and additional samples for 15 years.

#### Study population

Three phenotypic groups are identified on IHC done at inclusion: on metastatic sites or breast tumor if local recurrence, usual treatment protocols are often guided by the following groups:

- Group 1: Patients HR + (ER+ and/or PR+ and HER2-)

. patients with history of adjuvant therapy

. patients with de novo metastatic disease

- Group 2: Patients HER2 + with or without HR+

. patients with history of adjuvant therapy

. patients with de novo metastatic disease

- Group 3: Patients triple negative (HR- and HER2-)

- patients with history of adjuvant therapy.

- patients with de novo metastatic disease.

For statistical analysis we will define a specific subgroup of BRCA mutation patients studied to highlight specific elements according to the main objective.

#### Inclusion criteria

1. Written informed consent obtained from the patient prior to performing any protocol-related procedures, including screening biopsy, blood sample, faeces and questionnaires
2. Men or women > 18 years old at time of written consent
3. Patient with histologically confirmed breast cancer
4. Breast cancer metastatic disease or locally advanced not eligible for local curative treatment intent with or without personal history of adjuvant therapy for this cancer (chemotherapy, radiotherapy, surgery …)
5. Patient with metastases that can be biopsied.
6. Performance status ≤2 (according to WHO criteria)
7. Indication of any systemic therapeutic strategy can be performed alongside this current cohort in accordance with national and / or international recommendations.
8. HR and HER2 status on metastatic sites or breast tumor if local recurrence
9. Menopausal status: as per the institutional standard of care
10. Patient is willing and able to comply with the protocol for the duration of the study including undergoing treatment and scheduled visits and examinations including follow up.
11. Patient must be affiliated to a Social Health Insurance

#### Non-inclusion criteria

1. Other malignancy treated within the last 5 years (except non-melanoma skin cancer or in situ carcinoma of the cervix)
2. Coagulopathy or other pathology that contraindicates biopsy procedures
3. Prior systemic treatment in metastatic setting
4. Patients with exclusive brain metastasis not available for surgery
5. Pregnant or nursing patient
6. Individual deprived of liberty or placed under the authority of a tutor
7. Impossibility to submit to the medical follow-up of this clinical trial for geographical, social or psychological reasons

#### Study visits

Further evaluation visits (Table 1):

**Table 1:** Study visits – SPIRIT [20]

Study Period					
Assessments to be performed at the times stipulated in the table and as clinically required in the management of the patient	SCREENING ASSESSMENT	TREATMENT LINE (to be repeated at each progression)			DISEASE PROGRESSION VISIT
		Treatment	Early Evaluation	Evaluation visits	
Time point	0 to 4 weeks ± 7 days		After cycle 1	(h)	
Informed consent	X				
Eligibility screen	X				
Medical history + demographics	X				
Patient registration and enrollment	X(a)				
**PATIENT REPORTED OUTCOME (PRO)**					
Socio-demographic characteristics	X(a)			X(b)	
STAI TRAI	X				
STAI ETAT				X	X
QLQ-C30, QLQ-BR23, EDP,, BDI-II, physical activity questionnaire	X(a)			X	X
Food Frequency questionnaire	X(a)				X
Food inquiry	X(a; f)	X(f; g)			X(f)
PTGI				X(b)	
**CLINICAL EXAM**					
Physical exam, height, Weight, WHO performance status	X			X	X
AE/SAE assessment + Concomitant medications	X			X	X
Vital signs: temperature, respiratory rate, blood pressure, pulse, ECG	X			X	X
**BIOLOGY: STANDARD LABORATORY TESTS**					
Hematology, Coagulation parameters, Clinical chemistry, Thyroid function tests (1)	X			X	X
Disease-specific tumor markers (2)	X			X	X
Urine dipstick analysis (3)	X			X	X
βHCG (if applicable)	X				
**IMAGING EXAMS**					
Chest/Abdomen and Pelvis CT scan	X			X	X
^18^FDG PET/CT	X		X(c)	X(d)	X
Bone Scan	X			X(e)	X
**BIOLOGICAL AND BIOPATHOLOGICAL SAMPLING**					
Pathology: archived primary tumor sample	X				
Pathology: metastatic biopsy	X(a)				X
Biological blood samples: epigenetics biomarkers, cDNA, miRNA	X(a)		X	X	X
Biological blood samples: Circulating Tumor Cell - CTC	X(a)				X
Biological blood samples: Sphingolipids and extracellular vesicles	X(a)			X	X
Biological blood samples: proteomic analysis	X(a)				X
Urine sample: Molecular analysis	X(a)				X
Microbiota sample	X(a)	X(g)			X
**TREATMENTS**					
Treatment according to patient group		X			

a) After written informed consent signature

b) Once a year

c) Blinded assessment only

d) At first evaluation only

e) Only if bone metastasis at screening assessment

f) Food inquiry completed during 3 days: one week day, one weekend day and the day before microbiota sample

g) In case of diarrhea > grade 2 due to treatment

h) First evaluation visit:

Group1 without chemotherapy treatment to be done after 2 cycles

Group1, group2 and group 3 receiving chemotherapy treatment: the first evaluation visits are done 3 months and 6 months after treatment start (= end of chemotherapy)

For group1 without chemotherapy treatment: to be done every 4 months for 2 years, every 6 months for 3 years and once a year afterwards.

After end of chemotherapy: For group1, group2 group 3 receiving chemotherapy treatment: evaluation visits are to be done every 4 months afterwards.

1. Hematology: Hematocrit, hemoglobin, platelet counts, red blood cell counts, white blood cell counts, and white blood cell differential, Coagulation parameters: PT, PTT, INR, Chemistry: Albumin, Alkaline phosphatase, Alanine aminotransferase (ALT), Amylase, Phosphorus, Aspartate aminotransferase (AST), Bicarbonate, Calcium, Chloride, Creatinine, creatinine clearance, Gamma GT, Glucose, Lactate dehydrogenase (LDH), Lipase, Magnesium, Total protein, Potassium, Sodium, Total bilirubin, Direct Bilirubin, Conjugated Bilirubin, Uric acid, Total Cholesterol, Triglycerides, HDL, LDL, CRP, albumin, pré-albumin, orosomucoïde, Iron status: iron, ferritin, soluble transferrin receptor, Thyroid function: TSH and fT3 and fT4.
2. Disease-specific tumor markers: CA 15–3, ACE.
3. Urine dipstick analysis: Bilirubin, pH, Blood, Protein, Glucose, Specific gravity, Ketones, Colour and appearance (Microscopy should be used as appropriate to investigate white blood cells and use the high-power field for red blood cells).

## Data collection (Table 2)

### Data management

**Table 2:** EPICURE data producing units

Code	Data origin
EDPU-01	Biometrics (biological, clinical, epidemiological [environment omics], pathological)
EDPU-02	Circulating tumor cells
EDPU-03	Genomics
EDPU-04	Microbiomics
EDPU-05	Microbiomics - food survey
EDPU-06	Proteomics - bulk
EDPU-07	Psycho-oncology survey
EDPU-08	Radiomics - CT scan
EDPU-09	Radiomics - PET scan
EDPU-10	Transcriptomics - bulk
EDPU-11	Transcriptomics - single cell
EDPU-12	Treatment

Legend: *EPDU* EPICURE data producing unit, *CT* computed tomography, *PET* positron emission tomography

For clinical data management the platform used to collect and manage the database will be centralized and hosted with the entire control of the institution.

All access to all data (entry, modification or simple consultation) is only possible with a password and is plotted in the database.

According to the recommendations of regulatory authorities, procedures have been defined and implemented to ensure the physical and computer security of the data:

- Access is protected
- Equipment hosting the database are dedicated and deposited in a private bay of the secure data center.
- Backup of the computer system
- Measures ensure the safeguarding of the computer system
- Measures ensure the confidentiality of the data during the development of the computer application
- Measures ensure the confidentiality of data during the maintenance of software or equipment
- Authentication / Identification of the persons authorized to access the application

## Sample size and statistical analysis

Determination of sample size: the primary endpoint is to detect predictive factors (profile), based on clinical and molecular analyses, and associated with 5 years-overall survival.

With experience of observational studies in our institution like ESME [21], the accrual rate of patients meeting inclusion criteria is 165 patients per year. According to observed events (OS) occurring within 60 months of follow-up, the proportion of patients alive at 60 months is 15%.

To provide a power of 80% to detect a clinico- biological profile that reduced OS with a hazard ratio equal to 1.5 and to concede a 5% first species error rate alpha, we plan to include 300 patients. Indeed, the number of event would be around 254, which allow analysing 20 profiles.

For BRCA mutation we will collect this information to define a sub-group for specific analysis.

Statistical analysis process: Table 3

**Table 3:** Statistical analysis process

STEP 1	STEP 2	STEP 3
Collection of data: . Coordination in relation with clinical teams and clinical research units identified in the EPICURE project . Coordination of all platforms and units to avoid missing data	DATABASE PREPROCESSING: Use of efficient statistical tools for the reduction of the dimension in order to get *p* < 5000 at least for *n* = 300 patients: . Sparse Canonical Correlation analysis (in each class of variables) . Principal Component Analysis, Partial Least Squares	Mathematical development Adaptation of recent statistical methods of Data Mining to manage this high-dimension problem such as .LASSO/SLOPE methods (which select solutions with a weak number of « lighted » variables) and their variants adapted to the problem (Sparse Cox model to manage the censured data

## Discussion

The EPICURE study aims to prove feasibility of creation of a dynamic and longitudinal clinico-biological database prospectively by collecting epidemiological, socio-economic, clinical, biological, pathological, multi-omic data. It offers a systemic and “more exhaustive possible” approach to collect all data available without “a priori” on its interests and with large variety of data in the longitudinal way of real-life. Enrollment started in December 2018.

This cohort and its databases serve different research programs:

SIRIC-ILIAD project (Imaging and Longitudinal Investigations to Ameliorate Decision-making in Multiple Myeloma and Breast Cancer) on imaging and biological research approaches; program supported by INCA DGOS and INSERM.

FEDER program on molecular imaging technological approaches and data integration specific questions.

Several specific scientific projects; based yet on one or several clinical, biological or omic compartiment data.

## Data Availability

There are no data available as this is a study protocol.

## Abbreviations

CNRS: Centre National pour la Recherche Scientifique;
CRCINA: Centre de Recherche en Cancérologie Nantes Angers;
INSERM: Institut national de la Santé et recherché Médicale
BC: Breast Cancer
QoL: Quality of life
INCA: Institut National du Cancer
DGOS: Direction Générale de l’Offre de Soins
IHC: Immunohistochemistry
